# Elacestrant (RAD1901) exhibits anti-tumor activity in multiple ER+ breast cancer models resistant to CDK4/6 inhibitors

**DOI:** 10.1186/s13058-019-1230-0

**Published:** 2019-12-18

**Authors:** Hitisha K. Patel, Nianjun Tao, Kyung-Min Lee, Mariela Huerta, Heike Arlt, Tara Mullarkey, Steven Troy, Carlos L. Arteaga, Teeru Bihani

**Affiliations:** 1 Radius Health, Inc., 950 Winter St., Waltham, MA 02451 USA; 2 UT Southwestern Simmons Cancer Center, 5323 Harry Hines Blvd., Dallas, TX 75390 USA

**Keywords:** Elacestrant, RAD1901, SERD, CDK4/6, Resistance, Palbociclib, Ribociclib, Abemaciclib, Breast cancer, Estrogen receptor

## Abstract

**Background:**

Addition of CDK4/6 inhibitors (CDK4/6i) to endocrine therapy significantly increased progression-free survival, leading to their approval and incorporation into the metastatic breast cancer treatment paradigm. With these inhibitors being routinely used for patients with advanced estrogen receptor-positive (ER+) breast cancer, resistance to these agents and its impact on subsequent therapy needs to be understood. Considering the central role of ER in driving the growth of ER+ breast cancers, and thus endocrine agents being a mainstay in the treatment paradigm, the effects of prior CDK4/6i exposure on ER signaling and the relevance of ER-targeted therapy are important to investigate. The objective of this study was to evaluate the anti-tumor activity of elacestrant, a novel oral selective estrogen receptor degrader (SERD), in preclinical models of CDK4/6i resistance.

**Methods:**

Elacestrant was evaluated as a single agent, and in combination with alpelisib or everolimus, in multiple in vitro models and patient-derived xenografts that represent acquired and “de novo” CDK4/6i resistance.

**Results:**

Elacestrant demonstrated growth inhibition in cells resistant to all three approved CDK4/6i (palbociclib, abemaciclib, ribociclib) in both ESR1 wild-type and mutant backgrounds. Furthermore, we demonstrated that elacestrant, as a single agent and in combination, inhibited growth of patient-derived xenografts that have been derived from a patient previously treated with a CDK4/6i or exhibit de novo resistance to CDK4/6i. While the resistant lines demonstrate distinct alterations in cell cycle modulators, this did not affect elacestrant’s anti-tumor activity. In fact, we observe that elacestrant downregulates several key cell cycle players and halts cell cycle progression in vitro and in vivo.

**Conclusions:**

We demonstrate that breast cancer tumor cells continue to rely on ER signaling to drive tumor growth despite exposure to CDK4/6i inhibitors. Importantly, elacestrant can inhibit this ER-dependent growth despite previously reported mechanisms of CDK4/6i resistance observed such as Rb loss, CDK6 overexpression, upregulated cyclinE1 and E2F1, among others. These data provide a scientific rationale for the evaluation of elacestrant in a post-CDK4/6i patient population. Additionally, elacestrant may also serve as an endocrine backbone for rational combinations to combat resistance.

## Introduction

Breast cancer remains one of the most commonly diagnosed cancers in women and has affected over 200,000 women in the USA in 2018 alone [1, 2]. A majority (~ 70%) of breast cancers express the estrogen receptor (ER) and are driven by active ER signaling and corresponding transcription of genes that are important for tumor growth [2–5]. Current therapies for ER+ breast cancer rely heavily on their ability to block ER signaling either by inhibiting the synthesis of estradiol (aromatase inhibitors (AIs)) or by inhibiting ER signaling through competitive binding to the receptor itself [selective estrogen receptor modulators (SERMs) and selective estrogen receptor degraders (SERDs)] [6–9]. In the context of metastatic disease, dependence on active ER signaling is often maintained in metastatic lesions, leading to the continued use of endocrine agents such as AIs, tamoxifen (SERM), or fulvestrant (SERD) in this setting [10, 11].

While endocrine monotherapy has been a mainstay in treating metastatic ER+ breast cancer for decades, multiple pivotal clinical trials in recent years have demonstrated that the addition of cyclin-dependent kinase 4/6 inhibitors (CDK4/6i) to an endocrine agent, such as an aromatase inhibitor or a SERD such as fulvestrant, significantly increases progression-free survival (PFS) when compared to the endocrine agent alone [5, 12–17]. These trials led to the approval of the first-in-class CDK4/6i, palbociclib, followed closely by the approval of two additional CDK4/6 inhibitors, ribociclib and abemaciclib, both in the first-line and second-line settings for ER+ advanced breast cancer [18–20].

Recent overall survival (OS) data demonstrate that patients derive benefit from the combination of CDK4/6i with endocrine therapy. In patients with prior endocrine sensitivity in the PALOMA-3 trial, treatment with palbociclib and fulvestrant led to longer OS (10 months) than treatment with placebo and fulvestrant [21]. The MONALEESA-3 [22] and MONALEESA-7 trials demonstrated that both pre- and postmenopausal patients derived OS benefit from the addition of ribociclib to endocrine therapy, with a median OS not reached in either trial in the combo arm vs 40.0 months and 40.7 months, respectively, in the single-agent arms [23]. The MONARCH-2 trial demonstrated that treatment with abemaciclib plus fulvestrant resulted in a statistically significant median OS improvement of 9.4 months for patients who progressed after prior endocrine therapy regardless of menopausal status [24].

While the addition of a CDK4/6i to endocrine therapy approximately doubles PFS and improves OS, a portion of these patients will eventually relapse and will require additional treatment. It is important, therefore, to understand the molecular makeup of a tumor that has progressed on CDK4/6i treatment, in order to help inform subsequent treatment options. Several CDK4/6i resistance mechanisms have been described preclinically, including multiple alterations of the cell cycle pathway such as cyclin E1/E2 amplification, E2F1 overexpression, retinoblastoma protein (Rb) loss, and CDK6 overexpression driven by the loss of the tumor suppressor FAT1 among others [2, 25–28]. In the clinic, mutations in the Rb gene have been detected in patients after palbociclib treatment [29]; however, some studies suggest that these mutations are of low prevalence in breast cancer and are not likely to be a major mechanism of resistance [30–33]. While these mechanisms of resistance and their relevance in the clinic are currently being examined, a few key questions arise—Do these resistance mechanisms alter ER-driven tumor growth, and does an ER-targeted agent continue to be effective in patients with prior CDK4/6i therapy? Does the presence of constitutively active ESR1 mutations have an impact on the extent of ER-dependent tumor growth?

ER signaling is known to regulate several proteins that are important for cell cycle progression [5, 34, 35]. It has been previously demonstrated that ER regulates the expression of cyclin D1, a protein that complexes with CDK4/6, which in turn, phosphorylates and inactivates Rb, thus resulting in cell cycle progression [2, 34, 36]. Furthermore, ER is known to activate the expression of E2F1 which plays a major role in cell cycle progression through transcription of genes such as cyclin E1 [37, 38]. The impact that ER signaling has on genes heavily involved in cell cycle regulation may explain why the combination of antiestrogens and CDK4/6i is so effective. It also suggests that endocrine therapies will remain a mainstay in the treatment of breast cancer.

Given the impact of ER on cell cycle progression, it is important to evaluate antiestrogen therapy in a post-CDK4/6i setting. Fulvestrant, the only approved SERD, is effective in patients; however, its low oral bioavailability and intramuscular route of administration pose some limitations [39, 40]. This has spurred the discovery of new orally administered SERDs that can overcome these limitations. Herein, we assess the activity of elacestrant (RAD1901), an orally bioavailable SERD [41, 42], in CDK4/6i-resistant preclinical models. Elacestrant has demonstrated partial responses in patients with prior CDK4/6i therapy in phase I clinical trials (NCT02338349) [43]. Currently, elacestrant is being investigated in a phase III clinical trial (NCT03778931) in patients that have received prior CDK4/6i therapy [44]. Additionally, this will be among the first clinical trials to prospectively examine the effects of ESR1 mutations on response to hormonal therapies, which is important with respect to recent analyses demonstrating the selection of specific ESR1 mutations upon fulvestrant treatment [30]. We provide evidence demonstrating ER-driven tumor growth in a post-CDK4/6i tumor setting and preclinical rationale for the examination of elacestrant in patients that have progressed on a CDK4/6i.

## Materials and methods

### Reagents and cell lines

Elacestrant (RAD1901) ((6R)-6-(2-(N-(4-(2-(ethylamino)ethyl)benzyl)-N-ethylamino)-4-methoxyphenyl)5,6,7,8-tetrahydronaphthalen-2-ol dihydrochloride) was manufactured by Patheon. Elacestrant lots used in this study were periodically checked to ensure purity, stability, and chirality. HCC1428 cells were purchased from ATCC, and HCC1428-LTED (long-term estrogen deprived) were developed by maintaining the cells in RPMI phenol red-free medium supplemented with 10% charcoal-stripped FBS (HyClone, GE Healthcare Life Sciences) and 1% pen-strep (Thermo Fisher Scientific) at 5% CO_2_. MCF7 cells harboring wild-type ER were genetically modified using CRISPR-Cas9 to express either the *ESR1:Y537S* or the *ESR1:D538G* mutated proteins. Briefly, single-guide RNAs were utilized to create the KI/KI cell line containing *ESR1:Y537S* and the KI/KO cell line containing *ESR1:D538G*, and single-cell clones were isolated and grown to establish these cells. The MCF7-Y537S/D538G cell lines were maintained in RPMI phenol red-free medium supplemented with 10% charcoal-stripped FBS (HyClone, GE Healthcare Life Sciences) and 1% pen-strep (Thermo Fisher Scientific) at 5% CO_2_.

### Development of CDK4/6 inhibitor resistance

#### In vitro

HCC1428-LTED-Palbo^R^, HCC1428-LTED-Ribo^R^, and HCC1428-LTED-Abema^R^ cells were developed by exposing the HCC1428-LTED cells to increasing concentrations of the respective CDK4/6i to a final concentration of 500 nM for palbociclib, 1 μM for ribociclib, and 250 nM for abemaciclib. MCF7-LTED-Y537S-CDK4/6i^R^ and MCF7-LTED-D538G-CDK4/6i^R^ cell lines were developed by exposing MCF7-LTED-Y537S and MCF-LTED-D538G cells, respectively, to increasing concentrations of the respective CDK4/6i to a final concentration of 500 nM for palbociclib, 1 μM for ribociclib, and 250 nM for abemaciclib. Doses for generating resistant cell lines were chosen based upon the previously reported literature [25, 45, 46]. The ESR1 wild-type resistant cells exhibited a resistant phenotype after 10–12 months of drug exposure. The ESR1-mutant resistant cell lines exhibited a resistant phenotype after 6–10 months of drug exposure. After resistance was established, cells were maintained at 500 nM of palbociclib for Palbo^R^ cells,1 μM ribociclib for Ribo^R^ cells, and 250 nM of abemaciclib for Abema^R^ cells. MCF7 cells harboring wild-type ER were cultured in LTED conditions to serve as the control for the MCF7 cells harboring ESR1 mutations. The MCF7-WT-LTED cells lost ER expression (data not shown) and were not studied further. HCC-1428-LTED cells were used to understand CDK4/6 inhibitor resistance in a wild-type ER setting.

#### In vivo

ST941-HI (hormone-independent) patient-derived xenograft (PDX) fragments were implanted into ovariectomized athymic nude mice. Tumors were measured twice/week, with Vernier calipers; volumes were calculated using the following formula: (*L* × *W*^2^) × 0.5, where *L* is the length and *W* is the width in millimeters of the tumor. Once average tumor size reached 200 mm^3^, animals were either treated with vehicle, fulvestrant (3 mg/dose, weekly) + palbociclib (25 mg/kg, daily), or elacestrant (30 mg/kg, daily). Tumors growing in the presence of fulvestrant (3 mg/dose/week) + palbociclib (25 mg/kg daily) were allowed to grow at least 1500 mm^3^ and then harvested and re-implanted into a new cohort of mice considered as passage P1. This process was repeated to establish the subsequent passages (P2 and P3). The dose of palbociclib was reduced to 10 mg/kg in P3 to assess palbociclib activity at a clinically relevant dose (Additional file 1: Figure S3).

### In vitro cell proliferation assays

Briefly, HCC1428-LTED and HCC1428-LTED-CDK4/6i^R^ cell lines were seeded in 96-well plates at a density of 5000 cells/well. MCF7-LTED-Y537S, MCF7-LTED-Y537S-CDK4/6i^R^, MCF7-LTED-D538G, and MCF-7-LTED-D538G-CDK4/6i^R^ were seeded in 96-well plates at a density of 2000 cells/well. Twenty-four hours post-plating, cells were treated with the respective CDK4/6i or elacestrant. Cells were incubated in the indicated treatments for 7 days, and cell growth was measured using the CellTiter-Glo assay (Promega) as per the manufacturer’s instructions. Data was normalized to the control values as 100%, and data is graphed as the percentage of growth relative to control at day 7.

### Colony formation assay

**Fig. 1:**
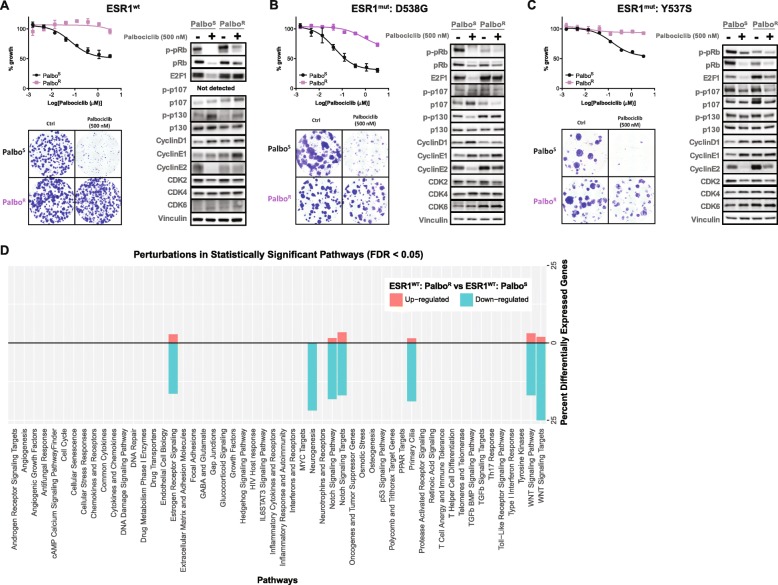
Characterization of palbociclib resistance models developed in ESR1 wild-type and ESR1 mutant (D538G and Y537S) backgrounds. CellTiter-Glo assay, colony formation assay, and western blot analysis of cell cycle proteins of **a** ESR1^wt^-Palbo^S^ and ESR1^wt^-Palbo^R^ cells, **b** ESR1^mut^: D538G-Palbo^S^ and ESR1^mut^: D538G-Palbo^R^ cells, and **c** ESR1^mut^: Y537S-Palbo^S^ and ESR1^mut^: Y537S-Palbo^R^ cells, treated with controls and palbociclib at the indicated doses. **d** Pathway perturbations in ESR1^wt^-Palbo^R^ vs ESR1^wt^-Palbo^S^ cell lines; red bar represents upregulated genes, blue bar represents downregulated genes

Cells were plated at a density of 1000–10,000 cells/well in 6-well plates. Twenty-four hours post-seeding, cells were treated with the indicated compounds [palbociclib (500 nM), ribociclib (500 nM), abemaciclib (250 nM), elacestrant (300 nM)]. Colonies were allowed to grow for 2–5 weeks depending on the growth rate of each cell line. The treatments were performed in triplicate, and media and drug were replaced weekly. At the end of the treatment, cells were fixed in paraformaldehyde and stained with 0.05% crystal violet for visualization. A representative well is pictured (Figs. 1 and 3).

### Western blotting

Cells were harvested and lysed in CelLytic^MT^ lysis buffer (Sigma-Aldrich) after either 24 or 48 h of treatment, and total protein was separated by SDS-PAGE and transferred to the membranes and immunostained using antibodies specific to the indicated proteins. For in vivo pharmacodynamic studies, end of study flash-frozen tumors (4 h post-last dose) were fractured using a cryoPREP instrument (Covaris), and pulverized tissue was lysed in CelLytic^MT^ lysis buffer (Sigma-Aldrich). Total protein was analyzed by Western blot analysis as described above. Protein expression was analyzed using standard practice and antibodies as follows: ERα, PR, E2F1, CCNE1, CCNE2, CCND1, total Rb, phospho-Rb S807/811, CDK2, CDK4, CDK6, Actin (Cell Signaling Technologies, Catalog #13258, #3153, #3742, #20808, #4132, #2978, #9309, #8516, #2546, #12790, #13331, #4970, respectively), phospho-p107, p107, phospho-p130, p130 (Abcam: ab111348, ab76255, ab168458, ab6545, respectively), GREB1 (Millipore, MAB562), and Vinculin (Sigma-Aldrich, #v9131). Protein expression was quantified using the AzureSpot software and normalized to the expression of the vinculin protein.

### Quantitative reverse transcriptase PCR analyses

For cell lines, quantitative reverse transcriptase PCR analysis was performed using the Cells to C_T_ kit (Life Technologies), and the lysates were processed according to the manufacturer’s instructions. qRT-PCR was performed using the one-step master mix and TaqMan™ probes (Applied Biosystems). For in vivo pharmacodynamic studies, end of study flash-frozen tumors were pulverized using the cryoPREP instrument (Covaris). From the pulverized tissue, total RNA was extracted using the RNeasy Mini Kit (Qiagen); qRT-PCR was performed using the TaqMan Fast Virus One-Step Master Mix and TaqMan™ probes (Applied Biosystems). The C_T_ values were analyzed to assess the relative changes in the expression of the TFF1 (trefoil factor 1/breast cancer estrogen-inducible protein; Hs00907239_m1), GREB1 (gene regulated by estrogen in breast cancer 1; Hs00536409_m1), and PGR (progesterone receptor; Hs01556702_m1) genes, with GAPDH (4310884E) as an internal control, using the 2^−ΔΔC_T_ method [47].

### RNA sequencing

RNA was extracted from HCC1428-LTED and HCC1428-LTED-Palbo^R^ cell lines using the RNeasy Mini Kit (Qiagen), according to the manufacturer’s instructions. The total RNA library was prepared by quantifying purified RNA by Qubit method and assessing RNA quality and intactness by the Agilent Bioanalyzer. All RNA samples were normalized to 100 ng for the library preparation. The resulting libraries were normalized to size-adjusted molarity of 2 nM. The samples were processed according to the TS RNA Access protocol and sequenced according to the standard sequencing protocol using 2 × 100 bp PE sequencing on an Illumina HiSeq sequencing platform. Raw reads quality control and clipping and trimming of sequences were performed by fastq-mcf. Cleaned reads were mapped to the human genome by STAR software v2.4. Pairwise differential expression testing was performed using Expression analysis (EA) Genomics’ Ensemble two group comparisons suite. In brief, EA’s Ensemble method summarizes the differential expression *p* values and classification probabilities from five popular tools—*t* test, limma, DESeq2, edgeR, and EBSeq—to produce a new *p* value for differential expression which has demonstrated proper type I error control and superior sensitivity. Aspects of each component test were utilized as input to a logistic regression model trained on data from The Cancer Genome Atlas (TCGA) which produces an estimate of the probability that a gene is differentially expressed between two conditions. This value is further transformed to a proper *p* value by comparison against its empirical cumulative distribution under the null established via bootstrap resampling of TCGA data from various cancer types.

### Patient-derived xenografts

All study protocols were reviewed by Radius, approved by Institutional Animal Care and Use Committees (IACUC), and conducted in accordance with the US and International regulations for the protection of laboratory animals. Female athymic nude mice (NU(NCr)-Foxn1nu or BALB/cAnNCrl-Foxn1nu) were obtained from Envigo RMS, Inc., Jackson Laboratories, Harlan Laboratories, or Charles River Laboratories and acclimated for 3 to 7 days prior to implantation. All mice were housed in pathogen-free housing in individually ventilated cages with sterilized and dust-free bedding cobs, access to sterilized food and water ad libitum, under a light-dark cycle (12–14 h circadian cycle of artificial light), and controlled room temperature and humidity. The WHIM43-HI PDX was derived and studied at Horizon (Saint Louis, MO). The ST941-HI and ST3932 PDX models were derived and studied at South Texas Accelerated Research Therapeutics (San Antonio, TX). The CTG-2308 and CTG-2432-HI PDX models were derived and studied at Champions Oncology (Rockville, MD). The PDX-R1 model was derived and studied at UT Southwestern Simmons Cancer Center. All animals were subcutaneously implanted with PDX models. When tumors grew to 150–200 mm^3^, mice were randomized based on tumor volume and administered the indicated treatments. Tumors were measured twice/week with Vernier calipers; volumes were calculated using the following formula: (*L* × *W*^2^) × 0.5, where *L* is the length and *W* is the width in millimeters of the tumor. Elacestrant, palbociclib, alpelisib (byl-719), and everolimus were administered orally and daily for the duration of the study. Preformulated, clinical-grade fulvestrant (Faslodex, manufactured by AstraZeneca) was obtained through third-party vendors and administered by subcutaneous injection once weekly. At the end of the study, tumors were harvested 4 h post-last dose unless otherwise indicated.

### In vivo pharmacokinetic analyses

The protocol for the animal experiment was approved by the Institutional Animal Care and Use Committee (IACUC) and conducted in accordance with the US and International regulations for the protection of laboratory animals. Athymic nude (FoxN1/NCR) mice of 6–8 weeks of age were provided by the Charles River Laboratories (Wilmington, MA). Mice were housed four per cage and fed 5060 (irradiated) chow from Lab Diet ad libitum*.* Prior to the start of the study, mice were randomized into groups based on the average body weight. Mice were given a single dose of palbociclib (formulated in 0.9% sodium chloride at 10 mg/ml) at either 2.5, 7.5, 25, or 75 mg/kg. Each animal was used for a total of four time points for blood collection. Time points for blood collection were 1, 2, 4, 6, 8, 12, 24, 36, 48, and 72 h. The blood was collected from the mandibular vein, and no more than 100 μl of blood was taken at a single time. The final blood collection was a terminal cardiac puncture. All blood was collected in K2-EDTA tubes. The whole blood was spun down for 10 min at 12,000 rpm to separate the plasma. The plasma was stored at − 20 °C for LC-MS analyses.

### Statistical analysis and data analysis

Statistical and graphical presentations were performed using GraphPad Prism 7. For cell proliferation assays, the IC_50_ was calculated by fitting a dose-response curve using a nonlinear regression model with a log(inhibitor) vs response curve fit. Relative IC_50_, determined as the concentration where 50% of the maximal response is observed, was calculated by the GraphPadPrism 7.0 curve fitting software. For all xenograft studies, body weights and tumor volumes were evaluated twice weekly. Tumors were measured twice/week with Vernier calipers; volumes were calculated using the following formula: (*L* × *W*^2^) × 0.5, where *L* is the length and *W* is the width in millimeters of the tumor. Tumor volumes were generally represented as mean ± SEM. Statistical evaluations of the differences between the groups were assessed using one-way ANOVA with Dunnett’s post-test. Percent tumor growth inhibition (%TGI) was calculated as [1 − (average relative tumor volume_treatment group_/average relative tumor volume_vehicle group_)] × 100.

## Results

### CDK4/6 inhibitors (CDK4/6i) exhibit differential activity in relevant breast cancer cells

The efficacy of the three CDK4/6i, palbociclib, ribociclib, and abemaciclib, was tested in breast cancer cell lines that represent an AI-resistant/long-term estrogen deprived (LTED) setting and harbor either wild-type (HCC1428-LTED, abbreviated as ESR1^wt^) or mutant (MCF7-LTED-D538G and MCF7-LTED-Y537S, abbreviated as ESR1^mut^: D538G and ESR1^mut^: Y537S, respectively) ER. These cells are herein referred to as “CDK4/6i-sensitive” cell lines. All three CDK4/6i significantly inhibited the proliferation of these cells with varied potency and extent of inhibition (Fig. 1, Additional file 1: Figure S1). In the short-term proliferation assay, abemaciclib exhibited the greatest potency and extent of growth inhibition among the three CDK4/6i, whereas the anti-proliferative effects of palbociclib and ribociclib varied depending on the cell line tested (Fig. 1, Additional file 1: Figure S1). Long-term ribociclib treatment of the CDK4/6i-sensitive cell lines only resulted in a partial growth inhibition in the colony formation assays, whereas treatment of cells with abemaciclib or palbociclib resulted in a more complete growth inhibition (Fig. 1, Additional file 1: Figure S1).

### Generation of CDK4/6i-resistant cells lines after long-term exposure

To model clinical resistance to CDK4/6i therapy preclinically, we exposed estrogen-independent, CDK4/6i-sensitive breast cancer cell lines described above to increasing concentrations of each of the three CDK4/6i over a period of 6–12 months. The cell lines that were not exposed to CDK4/6i [Palbo^S^ (palbociclib-sensitive), Ribo^S^ (ribociclib-sensitive), Abema^S^ (abemaciclib-sensitive)] and the cells that were exposed to CDK4/6i [Palbo^R^ (palbociclib-resistant), Ribo^R^ (ribociclib-resistant), Abema^R^ (abemaciclib-resistant)] were assessed in short- and long-term proliferation assays. The resistant cell lines exhibited decreased sensitivity to the respective CDK4/6i in both assays (Fig. 1, Additional file 1: Figure S1). A shift in the IC_50_, as well as a reduction in the extent of growth inhibition, was observed in the resistant lines upon treatment with the same CDK4/6 inhibitor. Growth inhibition in response to the drug was completely lost in some of the resistant cell lines, and in one case, the growth of ESR1^mut^: Y537S-Abema^R^ cells was stimulated by the presence of abemaciclib in the colony formation assay. Other cells selected for resistance, such as the ESR1^mut^: D538G-Abema^R^ cells, maintained sensitivity to abemaciclib treatment, albeit to a lesser extent when compared to their respective parental counterparts. Nevertheless, the observed shift in IC_50_, reduced extent of maximal growth inhibition, and the ability of cells to form colonies in the presence of the respective CDK4/6i marked a “resistant” phenotype (Fig. 1, Additional file 1: Figure S1).

### CDK4/6i-resistant models exhibit distinct changes in key cell cycle proteins

To identify the molecular mechanisms for the observed resistance, both the sensitive and the resistant cell lines were treated short term (24 h) with the corresponding CDK4/6i and compared to their respective controls (no treatment for the duration of assay). The observed molecular changes are summarized in Additional file 1: Table S1.

The most frequently observed finding was upregulation in the expression of E2F1 and its downstream target cyclin E1 (CCNE1) (Additional file 1: Table S1). Considering the role of E2F1 as a positive regulator of transcription and cyclin E1 as an important player in cell cycle progression, these observations fit the profile of cycling cells and are in line with the previous observations [25, 26]. Consistent with a study by Yang et al., marked decreases seen in E2F1 expression upon CDK4/6i treatment in the sensitive setting [25] were no longer observed in the resistant lines (Fig. 1, Additional file 1: Figure S1). We also observed phosphorylation of Rb despite CDK4/6i treatment of the resistant lines. These data suggest continued G1-S transition and cell cycle progression, demonstrating a loss of typical response to a CDK4/6i and an acquired “resistant” phenotype.

Modulation of Rb/Rb family members, cyclinsD1/E2, and cyclin-dependent kinases varied in the resistant cell lines, implying that these cells adapt differently to CDK4/6i exposure (Additional file 1: Table S1), although ultimately converging to an upregulated E2F1/CCNE1 axis. Trends that were observed included upregulation of p130 (in the ESR1^wt^ and ESR1^mut^: D538G lines), upregulation of CDK6 (in ESR1^mut^: D538G (except Ribo^R^) and ESR1^mut^: Y537S lines), and upregulation of cyclinD1 (in ESR1^mut^: Y537S lines). Interestingly, a trend towards increased expression of Rb (pRb), phosphorylated Rb (p-pRb) and its family members, and p107 and p130 in the ESR1^wt^-Ribo^R^ and ESR1^wt^-Abema^R^ cell lines was observed. CDK2 remained mostly unchanged while cyclin D1 and CDK4 showed trends of upregulation in the ESR1^mut^: Y537S cell lines. Cyclin E2 did not show a consistent trend of up- or downregulation in the resistant lines.

Apart from the changes in the expression of key players in the cell cycle pathway, global analysis of RNA sequencing data revealed that the ER pathway, the NOTCH pathway, and the Wnt pathway, among others, were significantly altered upon long-term palbociclib exposure of the ESR1^wt^ line (Fig. 1d).

### CDK4/6 inhibitor-resistant models retain ER and ER signaling

Modulation of ER and ER target genes upon CDK4/6i exposure observed by us (Fig. 1d), and others [25], led us to explore the impact of long-term CDK4/6i exposure on ER and downstream ER signaling targets [progesterone receptor (PR) and growth regulated by estrogen (GREB1)].

**Fig. 2:**
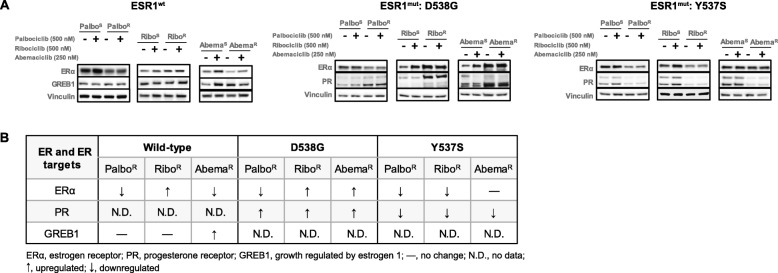
CDK4/6i-resistant cell lines retain ER and ER signaling. **a** Western blot analysis of ER and downstream ER target genes (GREB1/PR) in the CDK4/6i-sensitive and CDK4/6i-resistant ESR1 wild-type and ESR1 mutant cell lines. Palbo^S^/Palbo^R^, Ribo^S^/Ribo^R^, and Abema^S^/Abema^R^ cells were treated with either control or the indicated CDK4/6i for 24 h. **b** Table summarizing the changes in ER and ER target genes in the resistant cells when compared to the sensitive cell lines

In the ESR1^wt^-Palbo^R^ cells, a decrease in ER expression was observed; however, the expression of GREB1 remained unaltered. In the ESR1^wt^-Ribo^R^, the expression of ER and the target gene GREB1 remained unchanged. While ER protein levels were reduced in the ESR1^wt^-Abema^R^ cells, GREB1 expression was increased in comparison with the sensitive counterpart (Fig. 2). In the ESR1^mut^: D538G-Palbo^R^, downregulation of ER was observed; however, the expression of PR, an indicator of active ER signaling, was upregulated. In the ESR1^mut^: D538G-Ribo^R^ and ESR1^mut^: D538G-Abema^R^, an upregulation of ER and PR was observed. Overall, in the ESR1^mut^: D538G-CDK4/6i-resistant lines, PR expression was upregulated (Fig. 2). In contrast, in the ESR1^mut^: Y537S-Palbo^R^/Ribo^R^/Abema^R^, a consistent downregulation of PR was observed; however, changes in the ER expression in the resistant lines varied (Fig. 2).

Further evaluation of the ER pathway from the RNA sequencing data described above revealed upregulation of genes such as *EGR3* and *CAV1* and downregulation of other genes such as *PGR*, in the ESR1^wt^-Palbo^R^ cells, highlighting the importance of examining a complete ER-signature (Additional file 1: Figure S2A). The data taken together demonstrate that ER and ER signaling is maintained in models that have been exposed to CDK4/6i.

### Elacestrant inhibits growth of CDK4/6i sensitive and resistant lines

**Fig. 3:**
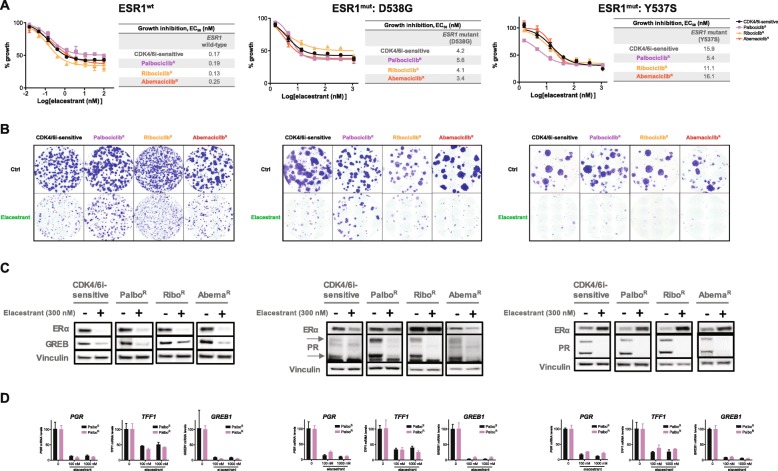
Elacestrant inhibits the growth of CDK4/6i-resistant breast cancer cell lines in vitro. **a** CellTiter-Glo assay of ER+ (wild-type and mutant) CDK4/6i-sensitive and CDK4/6i-resistant breast cancer cells treated with elacestrant at the indicated doses for 7 days. Relative EC_50_ values were calculated by using a log(inhibitor) vs response curve fit. **b** Colony formation assay of ER+ (wild-type and mutant) CDK4/6i-sensitive and CDK4/6i-resistant breast cancer cells treated with elacestrant at the indicated dose for 3–5 weeks. **c** Western blot analysis of ER and downstream ER target genes (GREB1/PR) in the CDK4/6i-sensitive and CDK4/6i-resistant ESR1 wild-type and ESR1 mutant cell lines. CDK4/6i-sensitive, Palbo^R^, Ribo^R^, and Abema^R^ cells were treated with either control or elacestrant (300 nM). **d** qRT-PCR of *PGR*, *TFF1*, and *GREB1* in palbociclib-sensitive and palbociclib-resistant cells treated with elacestrant at the specified doses

With ER and active ER signaling maintained in the CDK4/6i-resistant lines, the relevance of elacestrant treatment in a post-CDK4/6i setting was examined. The sensitive and resistant cell lines were treated with elacestrant, and proliferation was assessed in both a short-term (~ 7 days) and a long-term (3–5 weeks) cell assay. Elacestrant inhibited cell growth of the ESR1^wt^-CDK4/6i-sensitive and ESR1^wt^-resistant cells. The EC_50_ values and the extent of growth inhibition were similar regardless of the sensitivity or prior long-term exposure to each CDK4/6i (Fig. 3a). Additionally, despite each resistant cell line showing different levels of ER expression, the response to elacestrant was independent of which CDK4/6i was used to generate resistance (Fig. 3a). A similar observation was made in the ESR1^mut^: D538G and ESR1^mut^: Y537S sensitive and resistant cell lines (Fig. 3a). Overall, elacestrant inhibited the growth of these cells regardless of the sensitivity to CDK4/6i and which CDK4/6i they were resistant to. These growth-inhibitory effects were stable and maintained for long durations as demonstrated by the colony formation assays (Fig. 3b).

The effects of elacestrant on ER and ER signaling in the sensitive and resistant lines were assessed. Elacestrant degraded ER in the ESR1^wt^-CDK4/6i sensitive and resistant lines and downregulated GREB1 expression in all these cell lines. In the ESR1^mut^: D538G-CDK4/6i sensitive and resistant lines, a similar downregulation of ER expression was seen with a concurrent downregulation in PR expression. Elacestrant did not degrade ER in our genetically modified ESR1^mut^: Y537S cell lines; however, the downregulation of downstream ER signaling was still observed. Despite the varying effects on ER, a consistent downregulation of GREB1 or PR was observed across all the cell lines, indicating abrogation of ER signaling upon elacestrant treatment (Fig. 3c). Additionally, we examined mRNA levels of PR, TFF1, and GREB1 in all the lines to confirm that this inhibition of ER signaling was not limited to one downstream target. Indeed, we observed downregulation of PR, TFF1, and GREB1 upon elacestrant treatment in all the palbociclib-sensitive and the palbociclib-resistant lines (Fig. 3d). RNA sequencing data from the ESR1^wt^-Palbo^R^ cells demonstrated downregulation of additional ER targets, such as EGR3, upon elacestrant treatment (Additional file 1: Figure S2B).

Taken together, these data demonstrate the anti-tumor activity of elacestrant in clinically relevant CDK4/6i-resistant models in vitro, as well as inhibition of ER signaling and growth, regardless of sensitivity to CDK4/6i and ESR1 mutational status.

### Elacestrant inhibits growth of PDX models that represent CDK4/6i resistance

Elacestrant activity was examined in PDX models derived from patients that were either previously treated with and responded to a CDK4/6i or that exhibited de novo resistance to CDK4/6 inhibition. Additionally, a palbociclib-resistant PDX was developed in vivo to model acquired resistance to CDK4/6i in an in vivo setting.

**Fig. 4:**
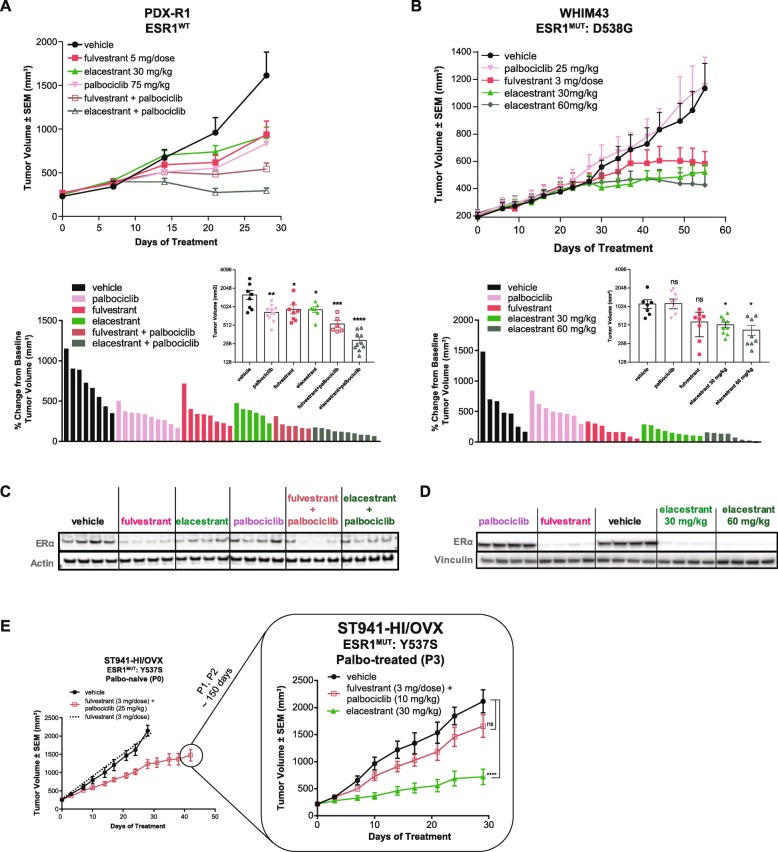
Elacestrant inhibits the growth of ER+ patient-derived xenograft (PDX) models that represent CDK4/6i resistance. Mean tumor volumes (*n* = 6–10/arm) ± SEM of PDX-R1 (**a**, top) and WHIM43 (**b**, top). Percent change in tumor volumes from baseline for individual tumors from PDX-R1 at the end of study (**a**, bottom) and WHIM43 at day 55 (**b**, bottom). For the PDX-R1 model, asterisks represent significant differences between drug-treated and vehicle-treated groups at the end of the study. For the WHIM43 model, statistical analysis of drug-treated groups vs vehicle-treated groups was performed on the day the vehicle-treated groups were taken down (day 55) (*p < 0.05, **p < 0.01, ***p < 0.001, ****p < 0.0001). **c** Western blot analysis of indicated proteins from tumors harvested 4 h post-last dose in the PDX-R1 model. **d** Western blot analysis of indicated proteins from tumors harvested 4 h post-last dose in the WHIM43 model. **e** Mean tumor volumes of ST941-HI PDX palbo-naive (P0) and ST941-HI PDX palbo-treated for > 150 days (P3) treated with a combination of fulvestrant and palbociclib, and single-agent elacestrant (in P3 only)

Elacestrant significantly inhibited the growth of PDX-R1 tumors harboring wild-type ER (Fig. 4a). A similar extent of growth inhibition was observed with fulvestrant. The PDX was derived from a patient who had progressive disease after being treated with letrozole in combination with palbociclib for about 14 months. Despite being derived from metastases that progressed on palbociclib, the PDX responded well to palbociclib (Fig. 4a). This could be due to the use of a higher dose of palbociclib than the human equivalent dose (Additional file 1: Figure S4), changes in the molecular characteristics of the tumor before PDX implantation, and/or the effect of subsequent treatments of the patient, with reversion of the tumor back to palbociclib sensitivity. The combination of palbociclib with elacestrant led to further tumor growth inhibition compared to the combination of palbociclib with fulvestrant (Fig. 4a). Degradation of ERα was observed upon treatment with single-agent elacestrant or fulvestrant, which was maintained upon the combination with palbociclib (Fig. 4c).

The WHIM43 PDX model harbors an ESR1-D538G mutation and has previously been demonstrated to be de novo resistant to palbociclib in vivo [48]. Consistent with this, the lack of Rb expression, a known mechanism of CDK4/6i resistance, has been reported for this model, which we also confirmed (Additional file 1: Figure S3). Elacestrant demonstrated significant tumor growth inhibition (TGI) at both doses tested while fulvestrant exhibited a non-statistical trend of growth inhibition; this trend did not reach significance when compared to the vehicle control (Fig. 4b). End-of-study tumor analysis revealed degradation of ER by both elacestrant and fulvestrant (Fig. 4d).

The ST941 model has been previously demonstrated to be insensitive to fulvestrant [49, 50]. We exposed this model to a combination of fulvestrant and palbociclib (P0) (Fig. 4e; fulvestrant data is extrapolated from a separate study) [49, 50]. Anti-tumor activity observed from the combination (Fig. 4e; left panel) is likely from palbociclib single-agent activity since the model has been shown to be insensitive to fulvestrant [49]. To model fulvestrant and palbociclib resistance, we re-implanted the tumors treated with the combination over two passages (P1, P2) for a total of > 150 days. In parallel, we assessed the pharmacokinetic properties of palbociclib to determine clinically relevant doses for the evaluation of resistance. In mice, clinically relevant exposure of palbociclib can be achieved at doses between 7.5 mg/kg and 10 mg/kg, and while 75 mg/kg or higher of palbociclib is often used in the literature, the exposure achieved at 75 mg/kg is likely not achieved in the clinic (~ 21× exposure of approved human dose; Additional file 1: Figure S4). Based on this, we reduced the palbociclib dose to 10 mg/kg for the last passage (P3). Elacestrant caused significant TGI in this model despite being exposed to fulvestrant and palbociclib for almost 6 months in vivo, indicating that these tumors retain ER-dependent tumor growth and sensitivity to elacestrant despite continuous exposure to the combination of fulvestrant and palbociclib (Fig. 4e).

Collectively, these data support the anti-tumor activity of elacestrant in models derived from patients that have been treated previously with a CDK4/6i (in combination with an AI/fulvestrant) and in PDX models that are innately resistant to CDK4/6i.

### Compensatory pathway inhibition in combination with endocrine therapy in a post-CDK4/6i setting

Investigation of combination therapies to combat resistance and target truncal drivers has been on the rise in the metastatic breast cancer setting. One such therapy that has been studied is the combination of alpelisib, a PI3Kα inhibitor, with endocrine agents such as letrozole or fulvestrant [51, 52]. The scientific rationale for this combination is based on the fact that mutations in the PIK3CA gene are frequently present (~ 30–40%) in breast cancer patients [53]. Additionally, a modest enrichment of PIK3CA mutations detected in ctDNA was observed in patients progressing on the combination of fulvestrant and palbociclib in the PALOMA-3 trial [30].

**Fig. 5:**
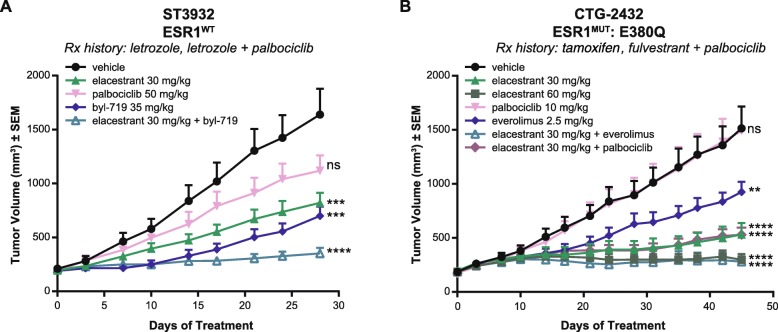
Elacestrant demonstrates anti-tumor activity as a single agent and in combination with PIK3CA pathway inhibitors. Mean tumor volumes (*n* = 10/arm) ± SEM of ST3932 (**a**) and CTG-2432 (**b**) PDX models in mice treated with indicated treatments. For the ST3932 model, asterisks represent significant differences between vehicle-treated and the indicated groups on the day the first vehicle-treated animal was taken down (day 28). For the CTG-2432 model, asterisks represent significant differences between the indicated groups at the end of the study. *p < 0.05, **p < 0.01, ***p < 0.001, ****p < 0.0001

We evaluated inhibitors of the PI3K/mTOR pathway in several PDX models harboring PIK3CA mutations derived from patients treated with a combination of an AI and palbociclib (ST3932 and CTG-2308) or fulvestrant and palbociclib (CTG-2432). The ST3932 model harbored an R88Q mutation in *PIK3CA* while the CTG-2308 models harbored an E545K mutation. The CTG-2432 model harbored an E545K and an E722K mutation in the PIK3CA gene and also harbors an E380Q mutation in *ESR1*. In the ST3932 model, palbociclib did not exhibit significant TGI (Fig. 5a), showing a lack of sensitivity to CDK4/6 inhibition, consistent with the lack of clinical response (Additional file 1: Figure S3B). Evaluation of single-agent elacestrant and the PIK3CA inhibitor alpelisib resulted in significant TGI, demonstrating active growth signals from both the ER and PI3K pathways (Fig. 5a). The combination of elacestrant and alpelisib led to complete TGI, supporting the use of inhibitors of the PI3K pathway in combination therapies in a post-CDK4/6i setting (Fig. 5a). In the CTG-2432 model, palbociclib did not inhibit growth at the clinically achievable dose of 10 mg/kg, demonstrating resistance to CDK4/6 inhibition. It is worthwhile to note that, like the WHIM43 model, analysis of this PDX revealed low levels or a lack of Rb expression when compared to other sensitive models (Additional file 1: Figure S3A). Single-agent elacestrant demonstrated significant TGI. Everolimus, an inhibitor of mTOR and a downstream effector of PI3K signaling, resulted in partial TGI as a single agent, albeit not to the extent of elacestrant. The combination of elacestrant (30 mg/kg) and everolimus led to further TGI (Fig. 5b).

In the CTG-2308 model, neither elacestrant nor fulvestrant resulted in significant TGI compared to the vehicle control (Additional file 1: Figure S5A). The sequencing data of this PDX model revealed the presence of a PIK3CA mutation (data not shown). Everolimus significantly inhibited the growth of this model, suggesting dependence on the PI3K-Akt-mTOR pathway for growth. Phosphorylation of S6, a downstream target of mTOR signaling, was significantly reduced in the everolimus single agent, and elacestrant + everolimus combination arms yet remained relatively unchanged in the elacestrant- or fulvestrant-treated arms (Additional file 1: Figure S5B). Examination of the tumors at the end of the study revealed that ER was degraded and ER signaling was inhibited by both fulvestrant and elacestrant in these tumors (Additional file 1: Figure S5C, S5D); however, this inhibition did not translate to TGI suggesting ER-independent tumor growth. This PDX was sensitive to palbociclib despite being derived from a patient that did not respond clinically to palbociclib (Additional file 1: Figure S3B). Phosphorylated Rb levels were significantly reduced in the palbociclib arms but were unaltered in the elacestrant or fulvestrant arms, which confirms the lack of ER-driven growth of this model.

Taken together, our data demonstrate the anti-tumor activity of elacestrant, as a single agent, and/or in combination with inhibitors of the PI3K/mTOR pathway, in models derived from patients who did not benefit from palbociclib treatment in the clinic.

### Elacestrant demonstrates anti-tumor activity in multiple CDK4/6i-resistant settings

**Fig. 6:**
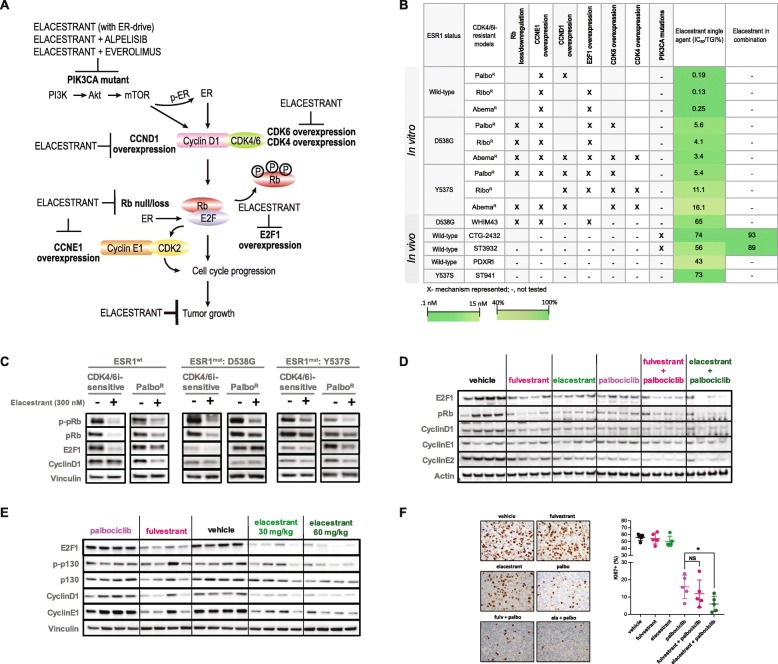
Elacestrant exhibits anti-tumor activity and downregulates key cell cycle proteins in multiple models of CDK4/6i resistance. **a** Pictorial representation of elacestrant activity in multiple models of CDK4/6i resistance. **b** Elacestrant activity, represented as IC_50_ and TGI, as a single agent and in combination in multiple models of CDK4/6i resistance. **c** Western blot analysis of indicated cell cycle proteins in in vitro models of palbociclib resistance. **d** Western blot analysis of indicated cell cycle proteins from tumors harvested 4 h post-last dose in the PDX-R1 model. **e** Western blot analysis of indicated cell cycle proteins from tumors harvested 4 h post-last dose in the WHIM43 model. **f** PDX tumors were harvested 4 h post-last dose in the PDX-R1 model. FFPE tumor sections were prepared and subjected to IHC with Ki67 antibody (Code# IR626, Agilent). The percent and their staining intensity were assessed by an expert breast pathologist blinded to the treatment to generate an *H*-score (*t* test). Representative images for each IHC were shown. Magnification, × 40

Our in vitro and in vivo PDX models exhibit distinct changes in key cell cycle markers upon continuous exposure to CDK4/6i (Additional file 1: Table S1). Despite the different contexts and molecular changes in each resistant derivative, elacestrant caused significant TGI (Fig. 6a, b), implying prior CDK4/6i treatment did not alter ER-dependent breast cancer cell growth and elacestrant activity in these contexts. In PDX models harboring PIK3CA mutations, the combination of elacestrant and inhibitors of the PI3K pathway led to further TGI (Fig. 6b).

Important cell cycle modulators that contribute to resistance, including those modulated by ER, such as cyclinD1 and E2F1, were examined after the elacestrant treatment in the in vitro and in vivo PDX models. In the ESR1^wt^ and ESR1^mut^-D538G/Y537S-Palbo^R^ lines, the downregulation of p-pRb was consistently seen across all cell lines after the elacestrant treatment, consistent with the growth inhibition observed. Elacestrant treatment resulted in a downregulation of E2F1 and/or cyclinD1 in most cell lines, suggesting that potent inhibition of ERα can, in turn, inhibit the cyclinD1/CDK4/6/Rb/E2F axis (Fig. 6c).

The observations made in the in vitro resistance lines translated to the long-term in vivo setting (Fig. 6d); the end-of-study analysis of PDX-R1 tumors demonstrated downregulation of E2F1, Rb, cyclinD1, and cyclin E2 after the elacestrant treatment. Similar observations were made in tumors treated with fulvestrant. The combinations of elacestrant or fulvestrant with palbociclib exhibited additional downregulation of E2F1 (Fig. 6d). Significant downregulation of Ki67 was also observed in the combination groups (Fig. 6f). Analogous results were seen in end-of-study tumors from the WHIM43 PDX model. Palbociclib-treated tumors demonstrated no changes in the expression levels of these proteins compared to the vehicle arm, supporting de novo palbociclib resistance seen in the model (Fig. 6e). Elacestrant- and fulvestrant-treated tumors demonstrated reduced expression of E2F1, cyclinD1, and cyclin E1. While the WHIM43 model is Rb-null, it maintains the expression of p130, a member of the Rb family. Elacestrant treatment resulted in reduced expression of phosphorylated p130. This suggests growth in the presence of palbociclib can be mediated by a compensatory family member in an Rb-null background and that elacestrant can inhibit the cell cycle through p130 (Fig. 6e).

Collectively, our data suggest that ER signaling is retained in most models insensitive to CDK4/6 inhibitors. We demonstrate in multiple models of CDK4/6i resistance that elacestrant exhibited anti-tumor activity and this activity was observed despite each resistant model exhibiting differential modulation of key cell cycle proteins. Additionally, elacestrant can serve as an endocrine backbone for PI3K/mTOR inhibitors in a post-CDK4/6i setting.

## Discussion

Herein, we evaluated the activity of elacestrant, a novel orally bioavailable SERD, in models that represent post-CDK4/6i or CDK4/6i-resistant patient populations. With CDK4/6i now a standard-of-care regimen in the mBC treatment setting, modeling CDK4/6i resistance in order to evaluate subsequent treatment options is vital. We comprehensively studied resistance to all three approved CDK4/6i in both ESR1-WT and mutant tumors. We demonstrate the anti-tumor activity of elacestrant in multiple in vitro models of acquired CDK4/6i resistance that harbored either wild-type or mutant ERα and retained ER-driven tumor growth. Additionally, this anti-tumor activity of elacestrant translates in vivo in multiple PDX models representing innate and acquired CDK4/6i resistance. This preclinical activity, along with the objective responses observed in patients with prior CDK4/6i therapy treated with elacestrant in a phase 1 clinical trial [43], suggests that ER-driven tumor growth can be retained in patients that have been previously treated with CDK4/6i and that the clinical evaluation of an ER-targeted agent, such as elacestrant, is warranted.

Palbociclib, ribociclib, and abemaciclib are currently approved for the treatment of metastatic breast cancer. While the kinome profiles of palbociclib and ribociclib have been shown to overlap to a great extent, abemaciclib has been suggested to target several other CDKs in addition to CDK4/6 [54], which could contribute to greater potency of growth inhibition observed in our models. Several studies have examined resistance to CDK4/6i, either through exposing cells to these inhibitors for months or by overexpressing kinases such as CDK6 to develop resistance [25, 26, 46]. Analogous to these studies, we note an increase in the expression of several cell cycle proteins that are important in the G1-S transition (Additional file 1: Table S1). Our data are consistent with previous preclinical reports of overexpression of cyclin E1, E2F1, cyclinD1, CDK4, and CDK6, and Rb loss, in models of CDK4/6i resistance [2, 25, 26, 55, 56]. Ma et al. demonstrated that E2F target genes, including cyclin E1, were significantly elevated in palbociclib-resistant patient tumors [57]. Gene expression analysis from baseline tumor tissues of the patients from the PALOMA-3 trial revealed that palbociclib efficacy was lower in patients who had higher *CCNE1* mRNA expression in metastatic tissue [58]. The authors demonstrated that the “E2F targets” hallmark gene set and high E2F transcriptional activity exhibited the most significant association with a lack of improvement in PFS from the palbociclib combination [58]. Our preclinical observations are in line with these clinical observations with the most-commonly observed mechanism of resistance being cyclin E1 overexpression, likely from increased E2F transcriptional activity (Additional file 1: Table S1). We also observed the overexpression of E2F1 in our models. The role of other players, such as the FAT1/Hippo pathway which is also known to mediate CDK4/6i resistance [28], is yet to be investigated. It is worth noting that we observe significant anti-tumor activity with elacestrant, even in models such as WHIM43, with increased cyclin E1 expression (Fig. 6b). In fact, high basal expression of cyclin E1 in the palbociclib-resistant WHIM43 PDX (Additional file 1: Figure S2) was reduced upon elacestrant treatment (Fig. 6e). While fulvestrant demonstrated a trend of growth inhibition in this model, this trend did not reach significance. Regardless of the mechanism employed by these tumor cells that leads to an upregulated CCNE1/E2F signature, elacestrant retained anti-tumor activity in in vitro and in in vivo PDX models (Figs. 2, 3, and 4).

ER plays a substantial role in the growth of breast cancer by regulation of cell cycle proteins, such as cyclinD1, E2F1, and c-myc, among others [37, 59–61], and tumor cells resistant to CDK4/6i continue to rely on the ER pathway to drive tumor growth. Indeed, we observed downregulation of cyclinD1, cyclin E1, and E2F1, upon elacestrant treatment in multiple representative models (Fig. 6). It has been previously demonstrated that ER antagonists elicit a distinct, non-overlapping cell cycle arrest program [62]. The growth inhibition activity of elacestrant being maintained in our in vitro models despite prior CDK4/6i exposure could be attributed to this distinct mechanism of cell cycle arrest. A recent retrospective analysis by Xi et al. demonstrated that hormonal therapy was effective, leading to significant PFS, in patients after palbociclib progression [63]. This supports our preclinical observations that ER signaling, and ER-mediated breast cancer cell growth, is maintained in a post-CDK4/6i setting and warrants the evaluation of elacestrant in patients after disease progression on a CDK4/6 inhibitor.

Higher IC_50_’s for ER-targeted agents such as tamoxifen and fulvestrant, and reduced ER and PR expression, have been noted in models of abemaciclib resistance that overexpress CDK6 [25]. While we similarly note increased CDK6 expression in our ESR1-mutant models, this did not confer diminished elacestrant antagonism, possibly due to alternate primary resistance mechanisms driving the growth of the cancer cells in our models. In general, ER signaling was maintained or increased in the ESR1^wt^ and ESR1^mut^: D538G lines. However, we do observe that not all PDX models derived from patients treated with palbociclib rely on ER signaling to proliferate (e.g., CTG-2308, Additional file 1: Figure S5). For these patients, combinations with targeted therapies might be warranted, and indeed, we demonstrate significant anti-tumor activity when elacestrant is combined with everolimus in the CTG-2308 model (Additional file 1: Figure S5).

Other sub-clonal populations that were detected in the PALOMA-3 trial included clones containing ESR1 mutations [30, 64]. Hotspot mutations in the ER ligand-binding domain (LBD), such as the D538G and Y537S, can provide tumor cells a growth advantage, suggesting that these ESR1 mutations, to an extent, may display innate resistance to CDK4/6 inhibitors. This concept has been supported by clinical observations of enrichment or continued selection of ESR1 mutations during combination therapy with palbociclib and letrozole [65]. Additionally, enrichment of the hallmark “E2F targets” pathway, shown to contribute to clinical resistance to palbociclib [58], has been observed in cell lines harboring the Y537S mutation [66]. This mutation has also been previously shown to be resistant to fulvestrant in preclinical reports [67], including our observations where some models that harbor this mutation demonstrate insensitivity to fulvestrant (Fig. 4e) [42, 50]. Recent clinical evidence confirms these preclinical observations, and indeed, emergence/selection of the Y537S mutation was observed in fulvestrant-treated patients in the PALOMA-3 trial [30]. While fulvestrant lacked efficacy in the ST941 Y537S-mutant model (Fig. 4e) [50], elacestrant significantly inhibited tumor growth. Reasons for this differential activity may include a distinct mechanism of action/PK properties and/or greater target inhibition for this mutation by elacestrant compared to fulvestrant. Moreover, elacestrant caused significant TGI despite 6+ months of prior treatment with the combination of fulvestrant and palbociclib (Fig. 4e). This suggests, that despite ER-driven tumor growth being maintained in a post-CDK4/6i setting, not all ER antagonists may be able to effectively inhibit growth.

A recent report demonstrated the emergence of Rb mutations in end-of-treatment samples in a small percentage of patients treated with palbociclib, suggesting the selection of these mutations during treatment [30]. The authors highlight the sub-clonal nature of these Rb mutations compared to parallel detection of much higher allele fractions of the PIK3CA mutation, suggesting the truncal nature of PIK3CA clones. PIK3CA mutations are found in ~ 30–40% of breast cancer patients [53, 68, 69]. In the SOLAR-1 trial, the addition of alpelisib, a PIK3CA inhibitor, to fulvestrant prolonged PFS [70]. Additionally, a triple combination of CDK4/6i, endocrine therapy, and a PIK3CA inhibitor is currently being evaluated (NCT02088684). Whether the emergence of Rb mutations or increased allele frequency of PIK3CA mutants contributes to CDK4/6i resistance in our models has yet to be determined. Regardless, we demonstrate that elacestrant inhibits growth in models with upregulated, downregulated Rb or Rb-null cells and is able to produce significant TGI in models with ER-driven tumor cell growth and PIK3CA mutations (Figs. 3, 4, and 5). Similar to the SOLAR-1 trial (NCT02437318) [70] and previous preclinical observations [26], combining an endocrine agent, such as elacestrant, with alpelisib in the ST3932 model harboring a PIK3CA mutation resulted in further TGI (Fig. 5).

The data presented here demonstrate that employing an ER-targeted therapy is a relevant strategy to evaluate in a post-CDK4/6i setting. Elacestrant, a novel orally bioavailable SERD, significantly inhibits ER-mediated growth in clinically relevant in vitro and patient-derived models of CDK4/6i resistance. Additionally, elacestrant can serve as an endocrine backbone to combination therapies that target known driver pathways. These data provide a scientific rationale for the clinical investigation of elacestrant in a post-CDK4/6i patient population irrespective of ESR1 mutational status.

## Conclusions

With CDK4/6 inhibitors being an integral component in the treatment of metastatic breast cancer, it has become increasingly important to understand the impact of these inhibitors on the molecular characteristics of the tumor and, subsequently, second- and third-line treatment options. We demonstrate in preclinical cell line and patient-derived xenograft models that ER signaling mediated breast cancer cell growth is maintained in most settings despite de novo or acquired resistance to CDK4/6 inhibitors. Elacestrant, an oral SERD, inhibits ER signaling and growth of these CDK4/6i-resistant breast tumor cells. Additionally, elacestrant can combine with approved inhibitors of alternate driver pathways to further inhibit breast cancer cell growth. These preclinical findings warrant the clinical investigation of elacestrant in patients after progression on a CDK4/6i.

## Supplementary information

**Additional file 1: Figure S1.** Characterization of CDK4/6i (Ribociclib and Abemaciclib) resistance models developed in ESR1^wt^ and ESR1^mut^: D538G/Y537S backgrounds. CellTiter-Glo assay, colony formation assay and western-blot analysis of (A) ESR1^wt^/ESR1^mut^: D538G/ESR1^mut^: Y537S-Ribo^S^ and ESR1^wt^/ESR1^mut^: D538G/ESR1^mut^: Y537S-Ribo^R^ cells and (B) ESR1^wt^/ESR1^mut^: D538G/ESR1^mut^: Y537S-Abema^S^ and ESR1^wt^/ESR1^mut^: D538G/ESR1^mut^: Y537S-Abema^R^ cells; treated with controls and the pertinent CDK4/6i at the indicated doses. **Figure S2.** Elacestrant downregulates upregulated ER pathway genes in palbociclib-resistant cells. A. Log2FC for genes modulated in the ER signaling pathway in ESR1^wt^-Palbo^R^ cell line vs ESR1^wt^-Palbo^S^ cell line. B. Log2FC for genes modulated by elacestrant (300 nM) treatment of ESR1^wt^-Palbo^R^ cells. **Figure S3.** Comparative expression of cell cycle proteins in palbociclib-sensitive and palbociclib-resistant in vitro and in vivo models. A. Western blot analysis of indicated proteins from baseline/vehicle-treated samples of the denoted palbociclib-sensitive and palbociclib-resistant models. B. Summarized treatment histories and palbociclib responses for the PDX models shown in this paper. **Figure S4.** Single-dose pharmacokinetic profile for palbociclib in non-tumor bearing mice. Mice were treated with the indicated doses of palbociclib and plasma collected at the indicated timepoints after single dose. Mean concentration ± SD of palbociclib is depicted (*n*=4/timepoint/dose). Area under the curve (AUC_0-inf_) was calculated and divided by the AUC_0-inf_ for the clinical regimen of palbociclib (Ibrance-125 mg). **Figure S5.** ER-independent growth of PDX model previously treated with AI + palbociclib. A. Mean tumor volume of CTG-2308 PDX model, asterisks represent differences between the indicated groups at the end-of-study; *p*-values *, *p*<0.05, **, *p*<0.01, ***, *p*<0.001, ****, *p*<0.0001. B. Western blot analysis of phospho-RB and RB from tumors harvested 4h post-last dose. C. Western-blot analysis of indicated proteins from tumors harvested 4h post-last dose for the indicated treatment arms. D. qRT-PCR analysis of *PGR*, *TFF1*, and *GREB1* in end-of-study tumors treated with the elacestrant and fulvestrant. **Table S1.** Commonly observed mechanisms upregulated in CDK4/6i-resistance models include CCNE1 and E2F1 overexpression. Summarized results from Fig. 2 demonstrating modulation of cell cycle proteins in our in vitro models resistant to palbociclib, ribociclib, and abemaciclib.

## Data Availability

The datasets used and/or analyzed during the current study are available from the corresponding author on reasonable request.

## Abbreviations

AI: Aromatase inhibitor
AUC: Area under the curve
ctDNA: Circulating tumor DNA
CDK2/4/6: Cyclin-dependent kinase 2/4/6
CDK4/6i: Cyclin-dependent kinase 4/6 inhibitors
EGR3: Early growth response 3
E2F: E2 factor
ER: Estrogen receptor
ESR1: Estrogen receptor alpha gene
GREB1: Growth regulated by estrogen in breast cancer 1
IHC: Immunohistochemistry
LBD: Ligand-binding domain
LTED: Long-term estrogen deprived
PALOMA-3: Palbociclib (PD-0332991) Combined with Fulvestrant In Hormone Receptor+ HER2-Metastatic Breast Cancer After Endocrine Failure
PDX: Patient-derived xenograft
PGR: Progesterone receptor
RT-qPCR: Quantitative reverse transcriptase polymerase chain reaction
pRb/Rb: Retinoblastoma protein
SERD: Selective estrogen receptor degrader
SERM: Selective estrogen receptor modulator
SOLAR-1: Study Assessing the Efficacy and Safety of Alpelisib Plus Fulvestrant in Men and Postmenopausal Women with Advanced Breast Cancer Which Progressed on or After Aromatase Inhibitor Treatment
*TFF1*: Trefoil factor 1
%TGI: Percent tumor growth inhibition
