# Supplementary material for: Elacestrant (RAD1901) exhibits anti-tumor activity in multiple ER+ breast cancer models resistant to CDK4/6 inhibitors
Source: Breast Cancer Res. 2019 Dec 18;21:146. doi: 10.1186/s13058-019-1230-0 (PMC6921513; doi:10.1186/s13058-019-1230-0)
Supplement: Supplementary file 1 — Additional file 1: Figure S1. Characterization of CDK4/6i (Ribociclib and Abemaciclib) resistance models developed in ESR1wt and ESR1mut: D538G/Y537S backgrounds. CellTiter-Glo assay, colony formation assay and western-blot analysis of (A) ESR1wt/ESR1mut: D538G/ESR1mut: Y537S-RiboS and ESR1wt/ESR1mut: D538G/ESR1mut: Y537S-RiboR cells and (B) ESR1wt/ESR1mut: D538G/ESR1mut: Y537S-AbemaS and ESR1wt/ESR1mut: D538G/ESR1mut: Y537S-AbemaR cells; treated with controls and the pertinent CDK4/6i at the indicated doses. Figure S2. Elacestrant downregulates upregulated ER pathway genes in palbociclib-resistant cells. A. Log2FC for genes modulated in the ER signaling pathway in ESR1wt-PalboR cell line vs ESR1wt-PalboS cell line. B. Log2FC for genes modulated by elacestrant (300 nM) treatment of ESR1wt-PalboR cells. Figure S3. Comparative expression of cell cycle proteins in palbociclib-sensitive and palbociclib-resistant in vitro and in vivo models. A. Western blot analysis of indicated proteins from baseline/vehicle-treated samples of the denoted palbociclib-sensitive and palbociclib-resistant models. B. Summarized treatment histories and palbociclib responses for the PDX models shown in this paper. Figure S4. Single-dose pharmacokinetic profile for palbociclib in non-tumor bearing mice. Mice were treated with the indicated doses of palbociclib and plasma collected at the indicated timepoints after single dose. Mean concentration ± SD of palbociclib is depicted (n=4/timepoint/dose). Area under the curve (AUC0-inf) was calculated and divided by the AUC0-inf for the clinical regimen of palbociclib (Ibrance-125 mg). Figure S5. ER-independent growth of PDX model previously treated with AI + palbociclib. A. Mean tumor volume of CTG-2308 PDX model, asterisks represent differences between the indicated groups at the end-of-study; p-values *, p<0.05, **, p<0.01, ***, p<0.001, ****, p<0.0001. B. Western blot analysis of phospho-RB and RB from tumors harvested 4h post-last dose. C. Weste [file 13058_2019_1230_MOESM1_ESM.pdf]

Supplementary Figure 1.

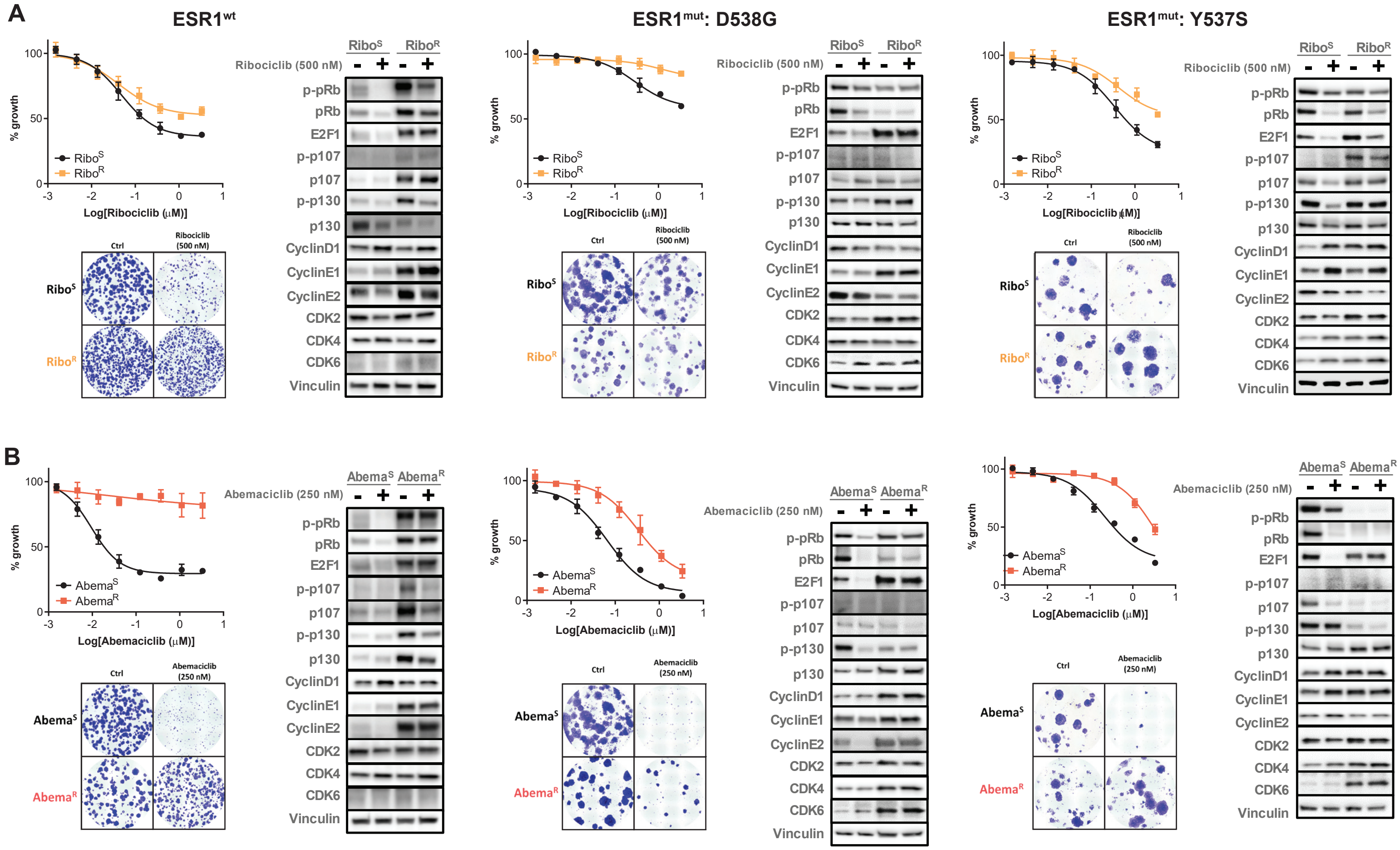

Supplementary Figure 2.

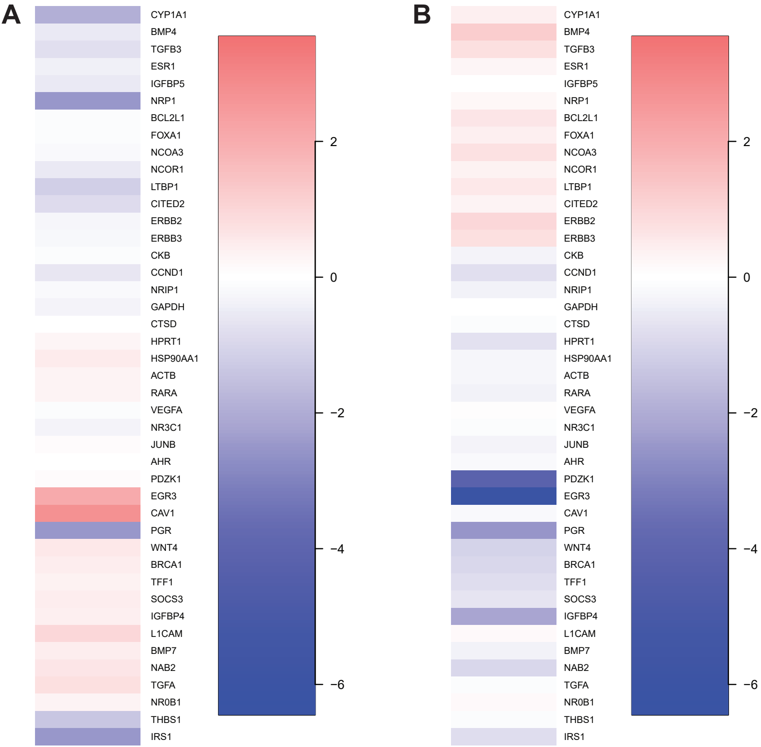

Supplementary Figure 3.

A

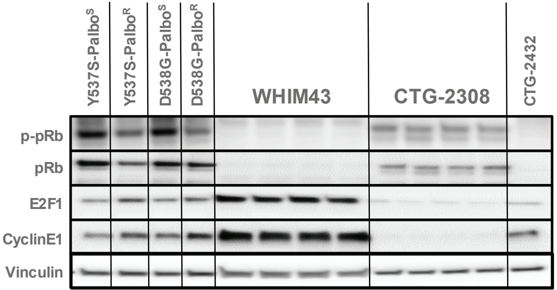

B

PDX	Treatment history	Palbociclib response in clinic	Palbociclib response in PDX model
WHIM43	Tamoxifen, AI, fulvestrant, chemotherapy (PDX collection)	not-treated	insensitive
CTG-2308	Letrozole, letrozole+palbo, chemotherapy (PDX collection)	no response	sensitive
CTG-2432	Tamoxifen, fulvestrant+palbo, chemotherapy (PDX collection)	no response	insensitive
PDX-R1	Tamoxifen, letrozole+palbo (PDX collection)	resistant after initial response	sensitive
ST3932	Chemotherapy, fulvestrant, letrozole+palbo (PDX collection)	no response	insensitive

Supplementary Figure 4.

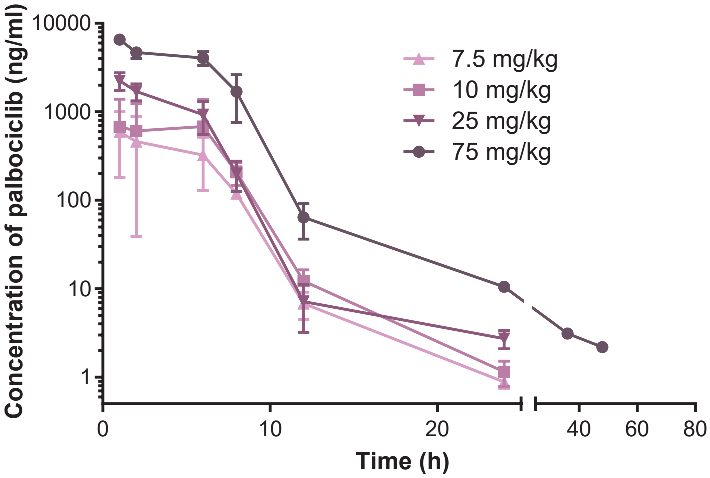

Palbociclib PK		
**Treatment**	**AUC_inf (ng·h/mL)**	**AUC_inf animal/human**
Palbociclib 7.5 mg/kg	2880	1.16
Palbociclib 10 mg/kg	4757	1.92
Palbociclib 25 mg/kg	8334	3.35
Palbociclib 75 mg/kg	53719	21.6
HUMAN Palbociclib 125 mg, single dose[1]	2483	n/a

[1]Tamura K, et al. *Cancer Sci*. 2016;107:755-63.

Supplementary Figure 5.

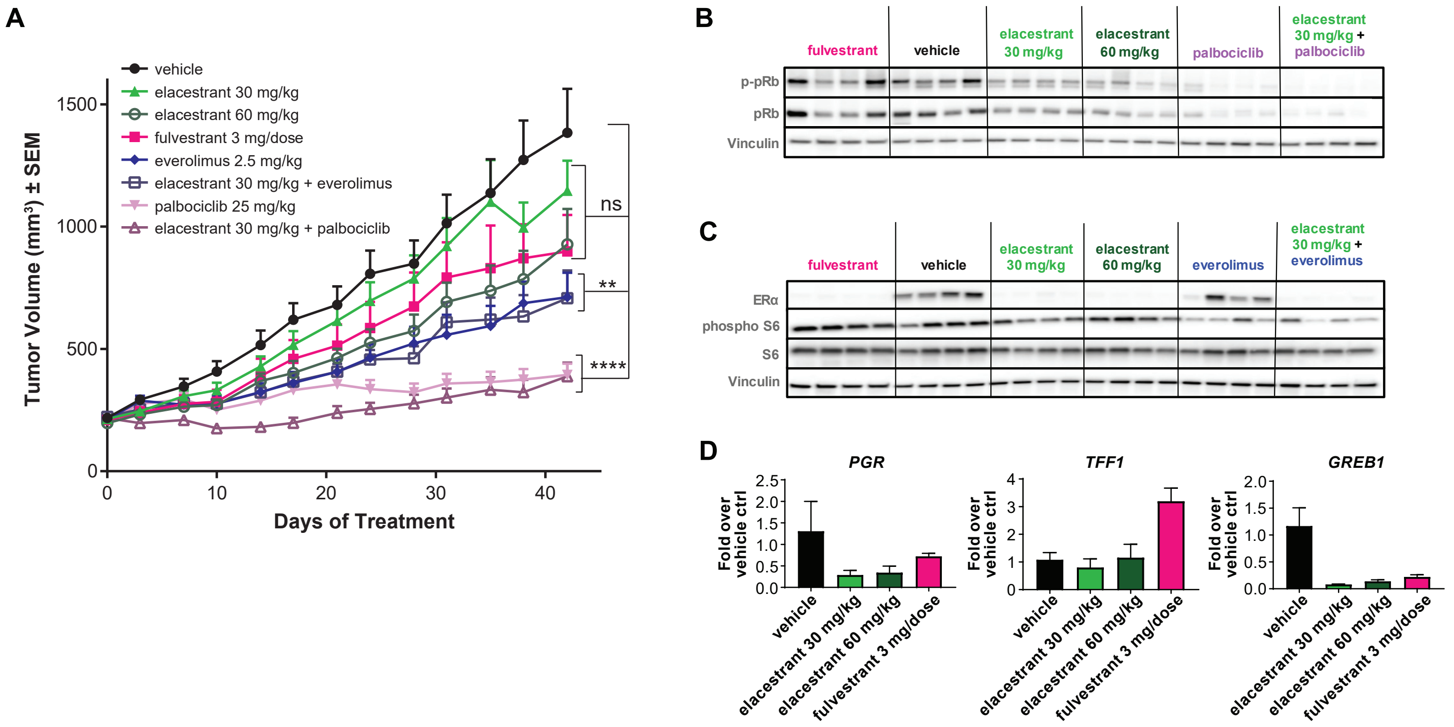

Supplementary Table 1.

CDK4/6i resistant cell line models		Rb and family members – transcriptional activators when phosphorylated						Trancription regulator	Cyclins – cell cycle transition			Cyclin-dependent kinases – cell cycle transition		
		pRb	p-pRb	p107	p-p107	p130	p-p130	E2F1	cyclin D1	cyclin E1	cyclin E2	CDK2	CDK4	CDK6
Wild-type	Palbo^{R}	N.C.	N.C.	↑	N.D.	N.C.	↓	N.C.	↑	↑	N.C.	N.C.	N.C.	N.D.
	Ribo^{R}	↑	↑	↑	↑	↑	↑	↑	N.C.	↑	↑	N.C.	N.C.	N.D.
	Abema^{R}	↑	↑	↑	↑	↑	↑	↑	N.C.	↑	↑	N.C.	N.C.	N.D.
D538G	Palbo^{R}	↓	↓	↓	N.D.	N.C.	↑	↑	↓	↑	↑	N.C.	N.C.	↑
	Ribo^{R}	↓	↓	N.C.	N.D.	N.C.	↑	↑	↓	↑	↓	↑	N.C.	N.C.
	Abema^{R}	↓	N.C.	N.C.	N.D.	↑	↑	↑	↑	↑	↑	N.C.	↑	↑
Y537S	Palbo^{R}	↓	↓	↑	N.C.	N.C.	N.C.	↑	↑	↑	↑	N.C.	N.C.	↑
	Ribo^{R}	N.C.	N.C.	N.C.	↑	N.C	N.C.	↑	↑	N.C.	N.C.	↑	↑	↑
	Abema^{R}	↓	↓	↓	N.D.	N.C.	↓	N.C.	↑	↑	N.C.	N.C.	↑	↑

↑, upregulated; ↓, downregulated; N.C., no change; N.D., not detected
